# Expression of SOX10 in Triple-Negative Breast Carcinoma in Pakistan

**DOI:** 10.7759/cureus.27938

**Published:** 2022-08-12

**Authors:** Seemal Ali, Zonaira Rathore, Zubaria Rafique, Akhtar S Chughtai, Aribah Atiq

**Affiliations:** 1 Histopathology, Chughtai Institute of Pathology, Lahore, PAK

**Keywords:** triple-negative breast carcinoma, sry-related hmg-box 10, sox10, invasive ductal carcinoma grade iii, invasive ductal carcinoma grade ii, tnbc

## Abstract

Background

The term triple-negative breast cancer (TNBC) refers to a particular class of aggressive, poorly differentiated neoplasms that show the absence of estrogen (ER), progesterone (PR), and human epidermal growth factor receptor 2 (HER2) antibodies. SOX10 (SRY-related HMG-box 10) is a nuclear transcription factor that is commonly used to identify cancers of neural origin, but it has recently been linked to TNBC. The purpose of this study is to determine SOX10 expression in TNBC, its association with tumor grade for molecular categorization, and to determine the diagnostic significance of SOX10 in TNBC at the metastatic site in the case of an unknown primary.

Methodology

SOX10 was used to stain a tissue microarray of 100 patients. According to the tumor grade, SOX10 staining was classified as negative (<1%), patchy (1-10%), focal (10-70%), and diffuse (70-100%). SPSS version 22 (IBM Corp., Armonk, NY, USA) software was used to conduct the statistical analysis.

Results

The expression of SOX10 regarding positivity and intensity was higher in high-grade tumors than in intermediate-grade tumors (p = 0.001 and p = 0.007, respectively).

Conclusions

SOX10 is a reliable novel marker for the diagnosis of TNBC and has diagnostic utility in the unknown primary at the metastatic site.

## Introduction

Globally, breast cancer is one of the most often diagnosed cancers in women of all ages [1] and affects one in eight women [2]. In Asia, Pakistan has the highest incidence of breast cancer, with one in nine women being affected in their lifetime [3]. Breast cancer is most common in women; however, only 6.6% of cases are seen in the younger age group of fewer than 40 years [4]. Many immunohistochemical (IHC) markers have been identified over the years to assist in the histological diagnosis of breast cancer. Among them, GATA-3 (GATA binding protein 3), GCDFP-15 (gross cystic disease fluid protein 15), and mammaglobin are most commonly used for the detection of primary and metastatic disease [5].

SOX10 (SRY-related HMG-box 10) is a new marker advised for breast cancer, which according to a few studies has shown promising results in diagnosis [6]. The possibility to target one or more of the genes that SOX10 controls as the basis for developing personalized therapy for metastatic breast cancer exists [7]. These genes are thought to be associated with aggressive breast cancer. Basal-like and unclassified triple-negative and metaplastic carcinomas demonstrate SOX10 expression [8].

Based on the results, we hypothesized that triple-negative breast cancer (TNBC) has a considerably greater SOX10 expression rate than other types of breast cancer. It might be employed as a potential biomarker for TNBC, which would aid in molecular classification. Therefore, this study aimed to assess SOX10 expression in TNBC to classify breast cancer, specifically metastatic TNBC, for pathological diagnosis in routine surgical specimens. To assess the prognostic significance of SOX10 in TNBC, the relationship between SOX10 expression and clinicopathological features of primary TNBC was also evaluated.

Several studies have been conducted at the international level; however, no data are available in our population regarding the expression of SOX10 in breast carcinoma. This study aimed to determine (i) the expression of SOX10 in TNBC as a diagnostic marker, (ii) its utility in the diagnosis of metastatic breast carcinoma in the case of unknown primary, and (iii) to determine its utility as a surrogate marker for molecular classification.

## Materials and methods

**Figure 1:**
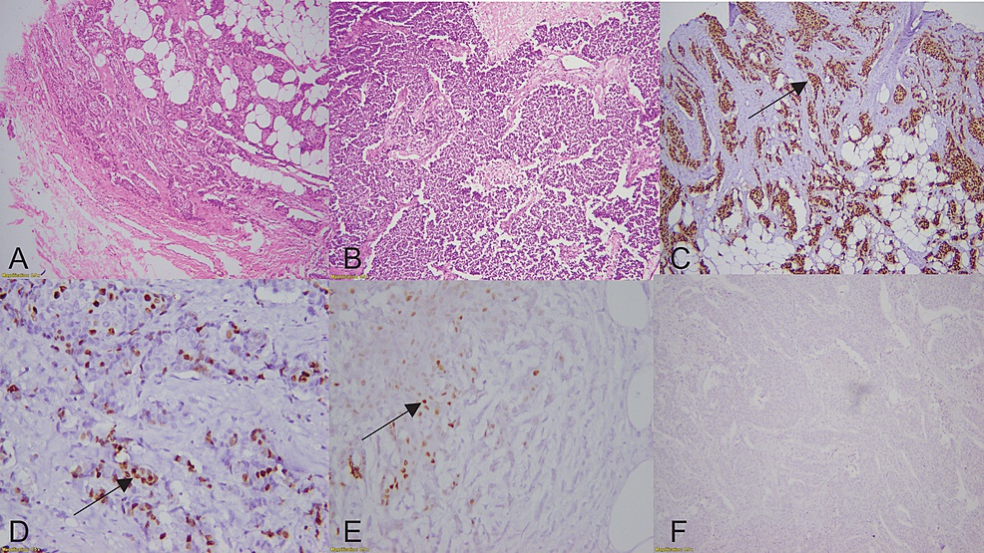
(A) H&E staining shows IDC grade II (100×) and (B) IDC grade III (×100). (C-F) showing staining pattern of SOX10 as (C) diffuse staining (black arrow), (D) focal staining (black arrow), (E) patchy staining pattern (black arrow), and (F) negative staining. H&E: hematoxylin and eosin; IDC: invasive ductal carcinoma

This cross-sectional descriptive investigation was carried out at the Chughtai Institute of Pathology, Lahore, Pakistan. All cases were retrieved from the archives of the Histopathology Department by using the electronic data system (Nexus) of the Chughtai Institute of Pathology between January 2019 and January 2021. Specimen collection was performed through tumor excision, incisional biopsy, and resection specimens. Primary diagnosis was made on hematoxylin and eosin (H&E)-stained sections. Two pathologists with a particular interest in this specialty reviewed 100 cases with TNBC in a blind manner. Using an automated tissue staining procedure (Peloris, Leica, Germany), IHC staining was performed on the sections with subsequent xylene dewaxing and ethanol rehydration. The tissue sections were heated at 100°C for 30 minutes in a citrate buffer to perform antigen retrieval. After 30 minutes at room temperature in 5% bovine serum albumin (BSA), the sections were counter-stained with hematoxylin, dried, and mounted before being examined under a microscope. The primary antibodies employed to categorize it as TNBC were estrogen (ER) (EP1 clone, monoclonal rabbit, ready to use), progesterone (PR) (PgR636 clone, monoclonal mouse, ready to use), and human epidermal growth factor receptor 2 (HER2) (polyclonal rabbit, diluted 1/300), all derived from DAKO (Glostrup, Denmark). After primary antibodies, SOX10 (EP268 clone, monoclonal rabbit, 1/500 dilution) derived from BIO SB (Goleta, California) was applied to determine its expression. Subsequently, the results were interpreted regarding the grade, pattern, and percentage of SOX10 staining in breast carcinoma (Figure 1).

Nuclear staining in more than 1% of cells was considered positive for SOX10. According to Allred scoring, more than 1% of tumor cells had ER and PR nuclear staining and more than 10% have complete circumferential HER2 membranous positivity was considered positive [9]. H&E slides with subsequent immunostained sections were used to obtain a consensual score. The criteria set for the interpretation of SOX10 were (1) negative (<1%), (2) patchy (1-10%), (3) focal (10-70%), and (4) diffuse (70-100%). Statistical evaluations were performed by applying SPSS software version 22 (IBM Corp., Armonk, NY, USA). The correlation between clinicopathological data and IHC expression was examined using Pearson’s correlation. Wherever required, Fisher’s extract test was applied. A p-value of 0.05 or less was considered statistically significant.

## Results

In our study, out of 100 TNBC cases, 32 were invasive ductal carcinoma (IDC) grade II (32%) and 68 were IDC grade III (68%). The age range was 19-70 years, with a mean of 44.31 ± 12.31 years. Both IDC grade II and grade III cases of TNBC showed distinct staining patterns with SOX10 as diffuse, focal, patchy, and negative staining.

In 32 cases of IDC grade II tumors, patchy staining was observed in six (18.7%), focal in seven (21.8%), and strong in only one (3.1%) case, while in 68 cases of IDC grade III TNBC, nine (13.2%) showed patchy, 25 (36.7%) showed focal, and 19 (27.9%) showed strong staining. However, 18 (56.2) cases of IDC grade II and 15 (22%) cases of IDC grade III TNBC showed negative staining (Table 1). Overall, out of 68 IDC grade III TNBC, 53 (61.9%) cases were positive for SOX10, and out of 32 IDC grade II tumors, only 14 (16%) showed positive staining for SOX10 (Table 1).

**Table 1:** Frequency of SOX10 staining pattern with the grade of the tumor (n = 100). SOX10: SRY-related HMG-box 10; IDC: invasive ductal carcinoma

Staining pattern of SOX10	Staining percentage of SOX10	Grade of tumor		Chi-square	P-value
		IDC II (n = 32) (%)	IDC III (n = 68) (%)		
Negative	<1%	18 (56.2%)	15 (22%)	24.2	0.0001
Patchy	1–10%	6 (18.7%)	9 (13.2%)		
Focal	11–70%	7 (21.8%)	25 (36.7%)		
Diffuse	71–100%	1 (3.1)	19 (27.9%)		
Total (n = 100)		32 (32%)	68 (68%)		

Overall, out of 68 IDC grade III TNBC cases, 53 (61.9%) were positive for SOX10, and out of 32 IDC grade II tumors, only 14 (16%) showed positive staining for SOX10 (Table 1).

The age, pathological grade, stage, and size of the tumor were correlated with SOX10 expression (Table 2). Only 62 patients had clinical information about the stage and size of the tumor.

**Table 2:** SOX10 expression with clinicopathological parameters (n = 100). SOX10: SRY-related HMG-box 10

Parameters		SOX10 expression		Chi-square	P-value
		Positive	Negative		
Age (n = 100)	<50	40	25	36.9	0.29
	≥50	23	12		
Grade (n = 100)	II	13	52	24.5	0.00006
	III	19	16		
Stage (n = 62)	II	28	11	0.922	0.619
	III	19	4		
Size (n = 62)	<5 cm	30	17	47.3	0.33
	≥5 cm	11	4		

SOX10 positivity and intensity were statistically significantly greater in high-grade tumors than in intermediate-grade tumors (p = 0.001 and p = 0.007, respectively). However, our study found no association between other clinicopathological features of primary triple-negative breast cancer, such as the age of onset, tumor size, or tumor stage (Table 2).

## Discussion

A growing number of women are devincreasinglting in an increase in the incidence of breast cancer. Risk factors for breast carcinoma include obesity, lack of physical activity, hormone replacement therapy, alcohol intake, early menarche, delayed first childbirth, and family history of breast cancer [10]. A few cases can also be associated with genetic mutations such as *BRCA1* and *BRCA2* [11]. According to the World Health Organization (WHO) categorization, there are approximately 20 major and 18 minor histologic subtypes [12]. Breast cancer has five main molecular subgroups that have been identified based on genetically determined tumor cell expression: luminal A, luminal B (HER2-negative or HER2-positive), HER2-enriched, and basal-like (triple-negative) [13]. These exhibit different molecular mechanisms that require different therapeutic management [14,15].

TNBC is a molecular subtype of breast cancer distinguished by the lack of specific biomarkers such as ER, PR, and HER2. A growing body of evidence suggests that TNBC represents 10-15% of all breast cancer cases [16].

Although various diagnostic methods have been used, tissue biopsy remains the gold standard for determining the diagnosis of any lesion, which is then validated by IHC stains and molecular investigations [17].

As shown in our study, SOX10 expression is associated with increasing grades. A similar study conducted in China by Linfang et al. showed concordant results [18]. Hence, SOX10 has promising utility in the diagnosis and can help in molecular classification [19]. However, when we encounter triple-negative breast cancer, particularly at metastatic sites, it is challenging to diagnose even with a particular clinical presentation. TNBCs are poorly differentiated high-grade tumors, requiring further confirmation by application of certain IHC stains, including GCDFP15, mammaglobin, and GATA-3 [5]. As the expression of SOX10 is known in melanoma and other tumors of other neural lineages, its expression in breast carcinoma is not explained in the literature and can be a pitfall for pathologists. The goal of this study is to emphasize that SOX10 is consistently positive in TNBC with increasing grade of tumor and in diagnosing unknown primary; hence, it can be helpful for pathologists to avoid misleading diagnoses at metastatic sites. SOX10 expression may lead to confusion in diagnosis but many studies have proven that breast carcinomas, particularly basal-like, triple-negative phenotypes, are also labeled by SOX10 [19]. This study is the continuation of a similar study conducted by Katharina et al. in Germany [20].

Breast carcinomas exhibit the greatest levels of SOX10 expression, which has been linked to stem-like properties and the promotion of mesenchymal transition [19]. The theory that TNBC derives from myoepithelial cells of the breast explains why SOX10 is expressed more in TNBC [21]. Numerous variables affect prognosis, including pathological grade, tumor size, staging, and lymph node metastasis. Poor prognosis is linked to high-grade tumors, high stage, and lymph node metastases [22]. Because the pathologic grade of primary TNBC in this study is correlated with SOX10 expression regarding intensity and pattern. This study found high-grade tumors had considerably high SOX10 expression (p = 0.001). Consequently, as a potential biomarker for TNBC treatment, SOX10 may be used to predict the prognosis and potential targeted genes. This supports our theory that increased SOX10 expression can be utilized as a tool for evaluating a patient’s prognosis for TNBC. Thus, detecting SOX10 expression using the IHC approach can be employed as an additional diagnostic marker for risk assessment.

Therefore, S0X10 can be a novel target gene for TNBC therapy and a new putative biomarker for assessing the prognosis and metastasis of primary TNBC.

## Conclusions

TNBCs are aggressive, poorly differentiated breast tumors that do not exhibit ER, PR, and HER2 antibodies. They can be challenging to diagnose at metastatic sites, especially in the case of an unknown primary. According to our preliminary investigation, SOX10 is a helpful marker for TNBC. Our study has shown that it can be used to diagnose primary breast cancer at the metastatic site and can be used for SOX10-targeted treatment for TNBC. Clinicians can concentrate on appropriate treatments by having a thorough understanding of the developing pattern of genetic alterations in breast cancer. More studies with a larger sample size are required to conclusively prove that these results emphasized the clinical implications for targeted therapy at the molecular level.

## References

[1] Mohapatra SK, Das PK, Nayak RB, Mishra A, Nayak B (2022). Diagnostic accuracy of mammography in characterizing breast masses using the 5th edition of BI-RADS: a retrospective study. Cancer Res Stat Treat.

[2] Filipits M, Nielsen TO, Rudas M (2014). The PAM50 risk-of-recurrence score predicts risk for late distant recurrence after endocrine therapy in postmenopausal women with endocrine-responsive early breast cancer. Clin Cancer Res.

[3] Khan NH, Duan SF, Wu DD, Ji XY (2021). Better reporting and awareness campaigns needed for breast cancer in Pakistani women. Cancer Manag Res.

[4] Assi HA, Khoury KE, Dbouk H, Khalil LE, Mouhieddine TH, El Saghir NS (2013). Epidemiology and prognosis of breast cancer in young women. J Thorac Dis.

[5] El Hag MI, Hag AM, Ha JP, Michael CW (2017). Comparison of GATA-3, mammaglobin, GCDFP-15 expression in breast carcinoma in serous effusions: a cell-block micro-array study. Pleura Peritoneum.

[6] Jamidi SK, Hu J, Aphivatanasiri C (2020). Sry-related high-mobility-group/HMG box 10 (SOX10) as a sensitive marker for triple-negative breast cancer. Histopathology.

[7] Saunus JM, De Luca XM, Northwood K (2022). Epigenome erosion and SOX10 drive neural crest phenotypic mimicry in triple-negative breast cancer. NPJ Breast Cancer.

[8] Feng W, Liu S, Zhu R, Li B, Zhu Z, Yang J, Song C (2017). SOX10 induced Nestin expression regulates cancer stem cell properties of TNBC cells. Biochem Biophys Res Commun.

[9] Ilić IR, Stojanović NM, Radulović NS (2019). The quantitative ER immunohistochemical analysis in breast cancer: detecting the 3 + 0, 4 + 0, and 5 + 0 Allred score cases. Medicina (Kaunas).

[10] Momenimovahed Z, Salehiniya H (2019). Epidemiological characteristics of and risk factors for breast cancer in the world. Breast Cancer (Dove Med Press).

[11] Shiovitz S, Korde LA (2015). Genetics of breast cancer: a topic in evolution. Ann Oncol.

[12] Lam SW, Jimenez CR, Boven E (2014). Breast cancer classification by proteomic technologies: current state of knowledge. Cancer Treat Rev.

[13] Eliyatkın N, Yalçın E, Zengel B, Aktaş S, Vardar E (2015). Molecular classification of breast carcinoma: from traditional, old-fashioned way to a new age, and a new way. J Breast Health.

[14] Aleskandarany MA, Vandenberghe ME, Marchiò C, Ellis IO, Sapino A, Rakha EA (2018). Tumour heterogeneity of breast cancer: from morphology to personalised medicine. Pathobiology.

[15] Venesio T, Siravegna G, Bardelli A, Sapino A (2018). Liquid biopsies for monitoring temporal genomic heterogeneity in breast and colon cancers. Pathobiology.

[16] Yin L, Duan JJ, Bian XW, Yu SC (2020). Triple-negative breast cancer molecular subtyping and treatment progress. Breast Cancer Res.

[17] Hoeve NDT, Moelans CB, Schrijver WA, Leng W de, van Diest PJ (2017). Molecular diagnostics in breast cancer routine practice. Eur Oncol Haematol.

[18] Jin L, Qin C, Qi X, Hong T, Yang X, Zhu X (2020). Clinicopathological significance of Sox10 expression in triple-negative breast carcinoma. Transl Cancer Res.

[19] Qi J, Hu Z, Xiao H (2020). SOX10 - a novel marker for the differential diagnosis of breast metaplastic squamous cell carcinoma. Cancer Manag Res.

[20] Kriegsmann K, Flechtenmacher C, Heil J (2020). Immunohistological expression of SOX-10 in triple-negative breast cancer: a descriptive analysis of 113 samples. Int J Mol Sci.

[21] Dravis C, Spike BT, Harrell JC (2015). Sox10 regulates stem/progenitor and mesenchymal cell states in mammary epithelial cells. Cell Rep.

[22] Narod SA (2012). Tumour size predicts long-term survival among women with lymph node-positive breast cancer. Curr Oncol.

